# Methotrexate efficacy, but not its intolerance, is associated with the dose and route of administration

**DOI:** 10.1186/s12969-016-0099-z

**Published:** 2016-06-14

**Authors:** J. Fráňová, Š. Fingerhutová, K. Kobrová, R. Srp, D. Němcová, J. Hoza, M. Uher, M. Saifridová, L. Linková, P. Doležalová

**Affiliations:** Paediatric Rheumatology Unit, Department of Paediatrics and Adolescent Medicine, General University Hospital and 1st Medical Faculty, Charles University in Prague, Ke Karlovu 2, Prague, Czech Republic; Paediatric Rheumatology Unit, Department of Paediatrics, Children´s Medical Center, Faculty of Medicine, Masaryk University Brno and Faculty Hospital Brno, Černopolní 9, Brno, Czech Republic; Institute of Biostatistics and Analyses at the Faculty of Medicine and the Faculty of Science of the Masaryk University, Brno, Czech Republic

**Keywords:** Juvenile idiopathic arthritis, Methotrexate, Efficacy, Intolerance, Toxicity, Quality of life

## Abstract

**Background:**

There is a lack of published evidence on the importance of methotrexate (MTX) dose and route of administration on both its efficacy and adverse events in children with Juvenile Idiopathic Arthritis (JIA). We aimed to document our clinical practice based on the treat-to-target approach in order to support the concept that better therapeutic effect achieved with an optimal dose of parenteral MTX is associated with clinically acceptable adverse effects comparable to those reported for oral treatment.

**Methods:**

Study inclusion criteria were indication of new MTX therapy for active arthritis in confirmed JIA patients younger than 18 years. Eligible patients were evaluated prospectively every 3 months for 1 year using standardized instruments for treatment response (American College of Rheumatology Pediatric (ACRPedi) response, Juvenile Arthritis Disease Activity Score (JADAS) 71, Clinically Inactive Disease (CID)) and adverse events (laboratory monitoring, Methotrexate Intolerance Severity Score (MISS)). MTX responders had to achieve at least ACRPedi 70 response. MTX intolerance was defined by MISS ≥ 6.

**Results:**

In 45/55 patients (81.8 %) MTX was started as subcutaneous injection. The initial median weekly dose was 14.4 mg/m^2^ in parenteral and 11.7 mg/m^2^ in oral administration. MTX therapy was effective in the level of ACRpedi70 and CID in 50.9 % and 30.9 % of patients at month 6 and in 70.9 % and 56.4 % after 12 months of the treatment, respectively. MTX intolerance at 6 and 12 months was noted in 25.5 % and 30.6 %, respectively. Management of intolerance included change in the dose and/or route of administration, education and councelling. Adverse events led to MTX withdrawal in 5 patients (9 %) due to toxicity (*n* = 3) and intolerance (*n* = 2). We did not find any significant predictive factors for either MTX therapeutic response or intolerance.

**Conclusion:**

Subcutaneous MTX weekly dose around 15 mg/m^2^ is associated not only with a high response rate within the first 12 months of treatment, but also with a relatively low rate of significant adverse effects that would lead to the treatment termination. It allows early recognition of MTX non-responders and addition of biologic therapy. Sustainability of therapeutic effect and longer-term evolution of adverse events will be addressed by an ongoing extension of the study.

## Background

Over the last decades low-dose weekly methotrexate (MTX) has been commonly used in the treatment of juvenile idiopathic arthritis (JIA) [1]. It has become an essential component of various clinical guidelines and recommendations as a first-line disease-modifying drug for disease unresponsive to nonsteroidals (NSAIDs) and/or intraarticular corticosteroids [2–4].

MTX efficacy has been reported mainly in polyarticular-course JIA, though its extent varies [5, 6]. Differences in drug dosing and route of administration as well as in the method of efficacy assessment hamper inter-study comparisons. Most commonly, American College of Rheumatology Pediatric (ACRpedi) response criteria have been used to define various levels of therapeutic effect [7]. More recently, a composite measure of disease activity (Juvenile Arthritis Disease Activity Index, JADAS) and its cut-off values for various disease status have been defined [8]. Among factors influencing inter-patient heterogeneity of treatment response, polymorphisms of genes involved in MTX metabolic pathways [9–15], disease phenotype and its previous duration and several biomarkers have been reported [16–20]. Moreover, factors related to MTX absorption and kinetics have also been shown to influence treatment efficacy [21–25].

Evaluation of the presence, type and severity of drug adverse events forms a natural part of therapeutic monitoring. Low-dose MTX has a variety of adverse effects. These include features of MTX antiproliferative effect which are mostly related to its post-dose concentrations [26–30]. MTX affects rapidly dividing cells of gastrointestinal (GI) tract causing nausea and/or vomiting and bone marrow leading to cytopenia. Mechanisms of hepatic and central nervous system (CNS) toxicity are more complex and include elevation of liver enzymes, headaches and behavioral changes [31, 32]. In addition to post-treatment complaints anticipatory and associative symptoms have been described [33–35]. Patients report presence of gastrointestinal symptoms or behavioral changes (cry, irritation, refusal to take the drug) already before or at the time of MTX administration (anticipatory intolerance) or even when just thinking about it (associative intolerance). They appear to be more commonly associated with parenteral route of MTX administration and often lead to precautious termination of otherwise safe and effective treatement [36, 37]. Therefore monitoring of MTX adverse events requires not only regular blood tests for hepatic and marrow toxicity, but also directed questioning to detect subjective complaints.

This study was driven by the lack of published evidence on the importance of MTX dose and route of administration on both its efficacy and various types of adverse events. We aimed to thoroughly document our clinical practice based on the treat-to-target approach in order to prove the concept that better therapeutic effect achieved with the higher dose of parenteral MTX is associated with clinically acceptable adverse effects comparable to those reported in published series using oral treatment.

## Methods

### Patients and study protocol

Consecutive patients were recruited prospectively from the paediatric rheumatology clinic population of the Department of Paediatrics and Adolescent Medicine, 1^st^ Faculty of Medicine, Charles University in Prague, between October 2013 and January 2015.

This was a sub-study of a large project running at the Unit aim of which was to prospectively collect clinical data on all consecutive JIA patients starting new treatments for active disease. The project was approved by the Local Research Ethics Committee of the General University Hospital in Prague and informed consent was obtained from the patients and/or their legal guardians, as appropriate. To become eligible patients must have had a definitive diagnosis of JIA according to the ILAR criteria [38] and exhibit active disease requiring either initiation of treatment for new-onset JIA or for the disease relapse. Active disease was defined by the presence of at least one joint with synovitis. Treatment options were: intra-articular triamcinolone hexacetonide, MTX, sulphasalazine, TNF-inhibitors and tocilizumab. At our Unit the treatment plan guided by the recently published ACR recommendations [2, 3] is always individually tailored to patient needs according to the treating physician. Only patients starting the MTX treatment are included in this report. The MTX therapeutic strategy is based on the following principles: 1. Early start, 2. MTX dose around 15 mg/m^2^/week administered parenterally using pre-filled syringes (MTX concentration 50 mg/ml), 3. Folic acid supplementation 5–10 mg once weekly 24–48 h after MTX dose, 4. The same dose which has induced disease inactivity is maintained for the minimum of 1 year if no clinically significant adverse events occur. Non-responders at 3–6 months change their treatment, usually by adding a biologic drug. Oral MTX in one weekly single dose is rarely considered in older children with relatively mild disease. Clinical and laboratory data and patient-reported outcomes are recorded within the interval of ± 2 weeks of the first MTX administration and then in 3-monthly intervals.

### Assessment of treatment efficacy

Treatment effect was evaluated by using two standardized methodologies for JIA activity assessment: ACR Pedi (The American College of Rheumatology) and JADAS (the Juvenile Arthritis Disease Activity Score). ACR Pedi uses the core-set of 6 disease activity variables (active joint count, limited joint count, physician global assessment of disease activity, parent/patient global assessment of the patient's well-being, Childhood Health Assessment Questionnaire and ESR/CRP) on 2 distinct occasions resulting in the defined improvement rates of ACR Pedi 30, 50, 70 and 90. These rates are defined as improvement of at least 3 out of 6 variables by a minimum of 30, 50, 70 or 90 % and no more than one of the resulting ones deteriorating by more than 30 % [7]. Good clinical response to MTX was defined as reaching the minimum of ACR Pedi 70. JADAS is a numeric score resulting from the values of 4 measures: active joint count (from 10, 27 or 71 joints), physician's global assessment of disease activity (10 cm VAS), parent/patient global assessment of overall well-being (10 cm VAS) and normalized ESR rate (0–10) [8]. JADAS-71 (score range 0–101) was chosen for this study. Additionally, disease state at 6 and 12 months was assessed according to the JADAS cutoff values as inactive disease (≤1) or minimally active disease (≤2 for oligoarthritis and ≤3.8 for polyarthritis) and high disease activity (4.2 for oligoarthritis and 10.5 for polyarthritis) [39–42].

Complete treatment response was also defined applying the Wallace criteria as the presence of inactive disease (clinically inactive disease, CID). CID is defined by an absence of active synovitis or other active JIA features (incl. uveitis, fever, rash, serositis, hepatosplenomegaly, lymhadenopathy attributable to JIA), normal ESR or CRP, the lowest possible physician global evaluation of disease activity and absence of significant morning stiffness (duration ≤15 min) [43–45].

### Assessment of MTX treatment adverse events

For the purpose of this study MTX adverse events were divided into toxicity and intolerance. Toxic adverse events were further assessed as measurable toxicity (increase of at least one liver transaminase above 2-times of upper normal limit (ULN) or significant cytopenia or anaemia) and patient-reported toxicity defined as patient complaints developing after MTX administration (gastrointestinal symptoms - abdominal pain, nausea, vomiting, oral ulcers, injection site reaction). Conditioned response to MTX-associated toxicity was defined as anticipatory intolerance when gastrointestinal (GI) complaints were present at the time of MTX administration, associative intolerance was considered when GI complaints occurred when thinking about MTX administration.

On top of that behavioral symptoms at the time of MTX administration were also recorded. Patient-reported symptoms were captured at every visit using the Czech adaptation of the Methotrexate Intolerance Severity Score (MISS), which defines MTX-intolerant patient by the presence of at least 6 out of 36 points with at least 1 point on anticipatory and/or associative and/or behavioral symptoms [34].

### Statistical analysis

Continuous and categorical variables were described using median with 25th and 75th percentile and relative frequencies. Mann–Whitney *U* test was used when assessing statistical significance of difference in continuous variables between two groups (when comparing more than two samples Kruskal-Wallis test with post-hoc Bonferroni correction was applied). Statistical significance of dependence between categorical variables was assessed with Fisher exact test. Time to treatment response was analyzed using Kaplan-Meier survival analysis method. Univariate logistic regression model was applied to measure association of baseline characteristics with occurrence of MTX intolerance (or treatment response). Level of statistical significance was set to 0.05 in all analyses.

## Results

### Characteristics of the patient cohort

Total of 107 patients fulfilled the study inclusion criteria during the 16-month recruitment period. In 58 children MTX therapy was initiated. Out of them 55 expressed their consent with the study participation. Their characteristics are summarized in Table 1. Their median age at study entry was 5.3 years (IQR 2.8;10.6), at disease onset 4.8 years (IQR 2.2; 10.0), disease duration was 3.8 months (IQR 2.5;6.4). The distribution of JIA ILAR onset subtypes at study entry was: oligoarthritis 38.2 % (*N* = 21) (persistent 32.7 % (*N* = 18), extended 5.5 % (*N* = 3)), polyarthritis 45.5 % (*N* = 25), enthesitis- related arthritis 5.5 % (*N* = 3), psoriatic arthritis 1.8 % (*N* = 1), systemic arthritis 9.1 % (*N* = 5). New onset disease was present in 48 patients (87.3 %), 7 patients had disease relapse. In these patients median interval from the previous medication withdrawal to the relapse was 10.5 months (IQR 6.0; 15.0). Median initial JADAS score was 12.0 (IQR 8.5; 20.2) reflecting active disease with the median number of joints with active arthritis of 5 (IQR 3; 6). High disease activity (JADAS cutoff values 4.2 for oligoarthritis and 10.5 for polyarthritis) was present in 40 children (72.7 %). Median JADAS in those who developed polyarticular course of the disease (regardless JIA onset subtype, *N* = 45) was 14 (ICQ 10.0; 20.4), patients with persistent oligoarthritis (at 12 months of follow-up, No =10) had median JADAS of 5.6 (ICQ 5.0; 8.5). Concomitant medication at the start of MTX therapy included systemic (oral) corticosteroids in 9 patients (16,4 %) (for systemic symptoms *n* = 4, resistant uveitis *n* = 3, severe polyarthritis *n* = 2) and intraarticular corticosteroids (within 1 month before or after MTX start) in 21 patients (38,2 %).

**Table 1:** Characteristics of 55 patients at study entry

Female n(%)	38 (69.1)
New onset n(%)	48 (87.3)
Age at onset (years) median (IQR)	4.8 (2.2; 10.0)
Age at MTX start (years) median (IQR)	5.3 (2.8; 10.6)
Interval from onset to MTX start (months) median (IQR)	3.8 (2.5; 6.4)
JIA subtype n	
– Oligoarthritis: persistent/extended	18/3 (32.7 %/5.5 %)
– Polyarthritis (RF positive n = 1)	25 (45.5 %)
– Systemic with polyarthritis	5 (9.1 %)
– Enthesitis-related	3 (5.5 %)
– Psoriatic	1(1.8 %)
Parent/patient global assessment of well-being mm, median (IQR)	27 (15; 40)
CHAQ 0–3 median (IQR)	0.25 (0.13; 0.69)
ESR mm/h median (IQR)	23 (15; 35)
MTX dose mg/m^2^ /week median (IQR)	14.2 (12.1;15.2)
– s.c. (n = 45)	14.4 (13.3; 15.9)
– p.o. (n = 10)	11.7 (8.3; 12.5)
Concomitant therapy n(%)	
– IATH^a^	21 (38.2)
– Sulphasalazine	1 (1.8)
– Systemic corticosteroids	9 (16.4)
– Folic acid 5–10 mg/week^b^	55 (100)

^a^Within 1 month prior to or after MTX start

^b^Once weekly dose 24–48 hours post-MTX administration

*MTX*, Methotrexate

*IATH*, Intraarticular triamcinolone hexacetonide

*CHAQ*, Childhood Health Assessment Questionnaire

Global assessments in mm on the 100 mm visual analogue scale

### MTX therapy

In 45 patients (81,8 %) MTX was started as once weekly subcutaneous injection, 10 patients received oral tablets (Table 1). There was no significant difference in demographic or disease parameters between the groups apart from the age at MTX start which was higher in patients starting oral treatment (median 14,0 years (IQR 11.8; 15.9) when compared to patients treated parenterally (median 4,1 years (IQR 2.6; 7.8) (*p* < 0,001). Parenteral MTX was administered in the median weekly dose of 14.4 mg/m^2^ (IQR 13.3; 15.9), oral MTX dose was 11.7 (8.3; 12.5) (*p* < 0,001).

Table 2 summarizes treatment changes, their timing and respective disease activity during the follow-up. MTX dose (median (IQR)) was increased from 12.1 (11.1; 13.7) mg/m^2^ at study entry to 14.9 (13.6; 15.6) and 13.8 (12.4; 15.8) mg/m^2^ at 6 and 12 months, respectively, in 18 patients (32.7 %). In 20 children (36.4 %) it was decreased from 14.3 (12.4; 16.1) to 13.7 (12.2; 15.2) and 10.5 (8.8; 12.8) mg/m^2^ at 6 and 12 months, respectively. Other treatment modifications included change in the route of administration (*n* = 13; 23.6 %), withdrawal (*n* = 5; 9.1 %), addition of biological therapy (*n* = 14; 25.5 %). Increased dose and change from oral to parenteral application were always due to the persistence of active disease. On the other hand, change from parenteral to oral administration and MTX dose reduction were triggered by combinations of MTX toxicity and intolerance on the background of the decreasing disease activity (Table 2). Treatment changes resulted in the median MTX dose of 14.2 mg/m^2^ (IQR 13.1;15.4) at 6 months and 13.7 mg/m^2^ (IQR 11.5;14.4) at 12 months for the whole cohort. At month 12 from 50 patients who were still receiving MTX only 9 had oral tablets.

**Table 2:** Changes in the treatment during the follow-up

	N (%)	Time from MTX onset (months)^a^	JADAS71 at the time of change^a^
↑MTX dose	18 (32.7)	3.0 (3.0; 6.0)	7.0 (4.5; 11.5)
↓MTX dose	20 (36.4)	7.5 (6.0; 9.0)	0.5 (0.0; 2.5)
Change in route of administration	13 (23.6)	6.0 (4.0; 12.0)	2.0 (0.4; 9.9)
Withdrawal	5 (9.1)	9.0 (8.0; 9.0)	3.0 (0.0; 6.5)
Addition of biologic	14 (25.5)	3.8 (4.0; 8.8)	15.8 (6.5; 13.0)

^a^values are medians (interquartile range)

All 14 patients who received biologic therapy either failed to achieve ACRpedi50 response (*n* = 11) and/or had resistant uveitis (*n* = 4). Their median JADAS71 at the time of treatment change was 15.8 (ICQ 6.50; 13.00). Addition of biologics (etanercept = 8, adalimumab = 4, tocilizumab = 2) occured after the median duration of MTX therapy of 3.8 (ICQ 4.0; 8.8) months.

### Treatment efficacy and disease activity assessments

MTX therapy was effective in the level of ACRpedi70 and 90 in 50.9 % and 42.9 % of patients at month 6 and in 70.9 % and 63.6 % after 12 months of the treatment, respectively. Clinically inactive disease was reached in 17 (30.9 %) patients at month 6 and in 31 (56.4 %) patients at month 12. There was no difference in the time to inactivity between various JIA subtypes. Patients who received biologics (*n* = 14) were considered non-responders to MTX from the time point of starting the biologic including 4 children who achieved CID after biologic medication was added to MTX who were excluded from the evaluation of disease inactivity. Total of 61.8 % patients reached low disease activity according to published JADAS cutoff values for oligo and polyarthritis (Table 3).

**Table 3:** Evolution of the treatment response expressed by different standardised measures

F/U (months)	ACRpedi70 (%)	ACRpedi90 (%)	JADAS inactive (%)	JADAS low activity (%)	CID (%)
3	28.6	12.7	5.5	21.8	9.1
6	50.9	42.9	32.7	47.2	30.9
9	56.4	50.9	43.6	54.5	47.2
12	70.9	63.6	56.4	61.8	56.4

*F/U*, Follow-up visit; *CID*, Clinically Inactive Disease [40]

Results for 55 patients are shown. Patients who received biologics and/or who withdrew MTX were considered non-responders from the time point of starting the biologic or stopping MTX

**Fig. 1:**
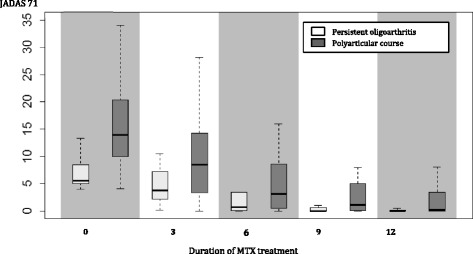
Treatment response expressed by JADAS in polyarticular-course and persistent oligoarticular JIA

Table 3 shows evolution of therapeutic response during 1-year follow-up as expressed by different disease activity measures. There was no significant difference in the proportion of patients who reached high levels of response when measured by either JADAS value (=0) or by fullfilling the CID criteria. Neither the rate or extent of therapeutic response were influenced by JIA onset subtype or by the route of MTX administration (data not shown). Figure 1 illustrates treatment response as expressed by JADAS71 values in persistent oligoarthritis and polyarticular-course JIA patients. Logistic regression analysis of demographic data, disease and treatment characteristics did not reveal any variable that would significantly influence the likelihood of therapeutic response (data not shown).

### MTX toxicity and intolerance

During the 12 month follow-up measurable toxicity of MTX was recorded in 8 patients (15.4 %): elevation of transaminases in 7 patients and cytopenia in 1 patient. In 3 cases these adverse events led to MTX withdrawal while in the remaining 5 children results normalised after the short treatment interruption or MTX dose reduction. None of the patients reported significant oral ulcers or injection site reaction.

Presence of patient-reported GI toxicity, associative/anticipatory intolerance and behavioral symptoms is summarized in Table 4. Only 11/55 patients (20 %) reported no adverse events throughout the observational period (MISS = 0 at every visit). Intolerance (MISS ≥ 6) slowly developed during the initial months of the treatment and was present in the maximum of 25.5 % of patients at 6 months and remained relatively stable thereafter (30.6 % at 12 months). This cut-off MISS value for the definition of intolerance was reached at least once in 25/55 children (45.5 %). Concomitant treatment with biologics did not have any significant impact on the MISS at 12 months (data not shown). In the majority of cases intolerance was managed to the patient and family satisfaction by treatment modifications and various other actions and their combinations: change in the route and/or timing of MTX administration, MTX dose reduction, addition of antiemetics, councelling. MTX withdrawal was the ultimate solution in 2 patients only (8 %).

**Table 4:** Evolution of gastrointestinal toxicity, anticipatory/associative intolerance and behavioral symptoms

Months	n^a^	MISS O	MTX intolerance	Anticipatory and/or associative symptoms	Gastrointestinal toxicity	Behavioral symptoms
		n (%)	n (%)	n (%)	n (%)	n (%)
3	53	19(3.8 %)	10 (18.5 %)	11 (20.8 %)	16 (30.2 %)	31 (58.5 %)
6	54	13 (24.1 %)	14 (25.5 %)	16 (29.6 %)	25 (46.3 %)	33 (66.1 %)
9	49	17 (34.7 %)	14 (28.0 %)	13 (26.5 %)	20 (40.8 %)	27 (55.1 %)
12	49	18 (36.7 %)	15 (30.6 %)	9 (18.4 %)	16 (32.7 %)	28 (57.1 %)

*MISS*, Methotrexate Intolerance Severity Score

^a^MISS not available for 2 patients at Month 3. In subsequent months numbers reduced by patients who withdrew (*n* = 5), in 1 patient MISS at 9 and 12 months not available

Behavioral symptoms were noted in at least one category in all intolerant patients, but they were present also in 27 (49 %) patients with the MISS lower than 6, regardless the route of MTX administration. The mean MTX dose in intolerant patients was not significantly different from that of tolerant ones (14.2 vs 13,4 mg/m^2^, OR (95 % CI) = 1,15(0.94;1,40), *p* = 0,612) (Table 5).

**Table 5:** Overall and per domain prevalence of MTX- related gastrointestinal and behavioral adverse events at 6 months

	All patients	MTX tolerant	MTX intolerant	Parenteral MTX	Oral MTX	*p*-value
		(MISS 0–5)	(MISS ≥ 6)			
Number of patients	*n* = 54	*n* = 40	*n* = 14	*n* = 47	*n* = 7	
Cutoff score ≥ 6	14 (25,9 %)	0 (0,0 %)	14 (100,0 %)	12 (25,5 %)	2 (28,6 %)	1
Cutoff score = 0	13 (24,1 %)	13 (32,5 %)	0 (0,0 %)	12 (25,5 %)	1 (14,3 %)	1
Anticipatory ± associative symptoms	16 (29,6 %)	7 (17,5 %)	9 (64,3 %)	14 (29,8 %)	2 (28,6 %)	1
Gastrointestinal toxicity	25 (46,3 %)	14 (35,0 %)	11 (78,6 %)	20 (42,6 %)	5 (71,4 %)	0,229
Behavioral symptoms	33 (61,1 %)	19 (47,5 %)	14 (100,0 %)	29 (61,7 %)	4 (57,1 %)	1
Abdominal pain	20 (37,0 %)	9 (22,5 %)	11 (78,6 %)	18 (38,3 %)	2 (28,6 %)	1
- after MTX	15 (27,8 %)	7 (17,5 %)	8 (57,1 %)	13 (27,7 %)	2 (28,6 %)	1
- anticipatory	6 (11,1 %)	2 (5,0 %)	4 (28,6 %)	6 (12,8 %)	0 (0,0 %)	1
- associative	4 (7,4 %)	1 (2,5 %)	3 (21,4 %)	4 (8,5 %)	0 (0,0 %)	1
Nausea	25 (46,3 %)	15 (37,5 %)	10 (71,4 %)	21 (44,7 %)	4 (57,1 %)	0,692
- after MTX	21 (38,9 %)	12 (30,0 %)	9 (64,3 %)	17 (36,2 %)	4 (57,1 %)	0,411
- anticipatory	8 (14,8 %)	1 (2,5 %)	7 (50,0 %)	8 (17,0 %)	0 (0,0 %)	0,577
- associative	9 (16,7 %)	4 (10,0 %)	5 (35,7 %)	7 (14,9 %)	2 (28,6 %)	0,33
Vomiting	5 (9,3 %)	2 (5,0 %)	3 (21,4 %)	5 (10,6 %)	0 (0,0 %)	1
- after MTX	5 (9,3 %)	2 (5,0 %)	3 (21,4 %)	5 (10,6 %)	0 (0,0 %)	1
- anticipatory	2 (3,7 %)	1 (2,5 %)	1 (7,1 %)	2 (4,3 %)	0 (0,0 %)	1
Behavioral symptoms	33 (61,1 %)	19 (47,5 %)	14 (100,0 %)	29 (61,7 %)	4 (57,1 %)	1
- restlessness	25 (46,3 %)	13 (32,5 %)	12 (85,7 %)	22 (46,8 %)	3 (42,9 %)	1
- crying	24 (44,4 %)	13 (32,5 %)	11 (78,6 %)	22 (46,8 %)	2 (28,6 %)	0,443
- irritability	21 (38,9 %)	8 (20,0 %)	13 (92,9 %)	19 (40,4 %)	2 (28,6 %)	0,693
- refusal of MTX	14 (25,9 %)	5 (12,5 %)	9 (64,3 %)	13 (27,7 %)	1 (14,3 %)	0,662

There were no serious adverse events (e.g. infections, pulmonary toxicity) that would require hospital admission throughout the study period. Logistic regression analysis did not demonstrate any significant associations of MTX intolerance with the patient, disease and treatment variables (Table 6). There was a trend towards increased odds for MTX intolerance in patients treated with parenteral MTX (OR (95 % CI) = 2,44 (0,56;10,65), *p* = 0,236), polyarticular-course of JIA (OR (95 % CI) = 1,43 (0,36; 5,78), *p* = 0,612) and higher age at MTX start (OR (95 % CI = 1,45 (0,48; 4,47), *p* = 0,505). Potential predictive factors of good MTX tolerance were higher CHAQ values, shorter interval from disease onset to treatment and male gender (Table 6).

**Table 6:** Potential predictors of MTX intolerance (MISS ≥ 6)

	MISS intolerance	
	OR (95 % CI)^a^	*p*-value
Male	0,70 (0,22; 2,22)	0,546
Age at onset (by 10 yrs)	1,61 (0,51; 5,02)	0,416
Age at MTX start (by 10 yrs)	1,46 (0,48; 4,47)	0,505
Interval from onset to MTX start (by 10 months)	0,41 (0,10; 1,58)	0,194
Uveitis before MTX treatment	0,87 (0,21; 3,67)	0,853
ANA positive	1,49 (0,51; 4,37)	0,465
Active joints (by 10 joints)	1,01 (0,55; 1,84)	0,979
Joints with limitation of motion (by10 joints)	1,12 (0,63; 1,98)	0,695
Physician global assessment of disease activity (by 10 mm)	1,22 (0,87; 1,69)	0,245
Parent/patient global assessment of well-being (by 10 mm)	0,88 (0,68; 1,14)	0,328
FW (by 10 mm/h)	1,26 (0,95; 1,67)	0,111
CRP (by 10 mg/l)	1,09 (0,89; 1,34)	0,409
CHAQ	0,30 (0,08; 1,17)	0,083
JADAS 71(á 10 points)	1,12 (0,68; 1,82)	0,659
Parenteral form of methotrexate	2,44 (0,56; 10,65)	0,236
Initial MTX dose (mg/m2)	1,15 (0,94; 1,40)	0,181
Polyarticular form of JIA	1,43 (0,36; 5,78)	0,612

^a^OR = odds ratio, 95 % IS = 95 % confidence interval

## Discussion

We have reported disease outcome in a single-centre cohort of patients with early JIA (median disease duration 3.8 months) during the first 12 months of MTX treatment. This prospective observational study was triggered by the lack of published evidence on the advantages of the parenteral versus oral route of MTX administration during the induction of remission therapy of JIA. Notably, we documented treatment efficacy using up-to-date standard disease assessments along systematic evaluation of MTX adverse events including conditioned effects like associative and anticipatory intolerance. We have shown that subcutaneous MTX weekly dose around 15 mg/m^2^ is associated not only with a response rate of ACRpedi 70 in over 70 % of patients, but also with a very low rate of significant adverse effects that would lead to the treatment termination.

An advent of cytokine inhibitors as well as better handling of standard JIA treatments like MTX have shifted our therapeutic target from reduction of disease activity to its complete elimination defined as disease inactivity and remission [46, 47]. With the constantly growing body of evidence on the biology of chronic arthritis it has become evident that time plays an important role in our ability to induce disease inactivity: The shorter disease duration before treatment onset and the more intensive therapeutic regimen used, the bigger is the chance to achieve sustained remission [19, 46, 47]. Such a treat-to-target approach has changed the way we use MTX now from the slow dose-escalation regime to the more aggressive treatment using the optimal effective dose established by Ruperto et al. at 15 mg/m^2^ [33] from the very beginning. Moreover, pharmacokinetic studies suggesting better bioavailability of parenteral against oral MTX [25, 26] have led us to starting MTX treatment in majority of patients in the form of subcutaneous injections. This approach allows for early assessment of the patient's potential to respond to treatment and to introduce biologic therapy in non-responders as early as within 3–6 months from starting MTX.

Indeed, in our cohort of 55 patients starting MTX only 10 (18.2 %) received oral tablets. The difference in the initial MTX dose and route of administration from some recently published series is illustrated in Table 7. Treatment response at 6 and/or 12 months is expressed by the percentage of patients achieving ACRpedi70 and/or CID. Proportion of MTX responders at 6 months is similar across most of the studies ranging from 38 to 56 % of patients [33, 44–53], similar to our results. Only a few studies provide information on the rate of inactive disease [43]. The higher rate of CID in our cohort (30.9 %) at 6 months when compared to the studies by Ruperto et al. (12 %) [33] and Wallace et al. (23.3 %) [43] could be explained by higher MTX dose and shorter prior disease duration in our patients. Although the dose of 15 mg/m^2^ is close to 0.5 mg/kg in older children, dosing per body weight unit in younger individuals ends up with a lower calculated dose. We believe these reasons together with the parenteral route of MTX administration apply also to the higher rate of responders in our cohort after 1 year of parenteral MTX therapy which exceeded 70 % of patients with the total of 56.4 % reaching CID (Table 3). Additionally, the variability of results in different studies also reflects differences in their design (retrospective analysis from the registry, prospective observational, prospective treatment trial).

**Table 7:** MTX efficacy in relation to prior disease duration, dose and route of administration

Publication	No of patients	Prior disease duration	MTX dose (route)	ACRpedi70 (%)/CID (%)	
				Treatment duration (months)	
				6	12
Ruperto 2004 [33]	595	2.7 yrs (mean)	10 ± 2.3 mg/m^2^ (78 % p.o.)	38/12^b^	
	40		14.5 ± 1.3 mg/m^2^ (s.c., i.m.)^a^		45/12.5^b^
	40		28.5 ± 2.5 mg/m^2^ (s.c., i.m.)^a^		47.5/10^b^
Bartoli 2007 [61]	125	1.45 yrs (med)	10 mg/m^2^ (NA)	26.4/NA	
Tynjala 2011 [62]^c^	20	1.5 mo (mean)	15–30 mg/m^2^ (p.o.,s.c.)		60/25
Klein 2012 [50]	259	1.1 yrs (med)	0,4 mg/kg (p.o.)	51/NA	66/NA
	152	0.8 yrs (med)	0.42 mg/kg (s.c.)	53/NA	
Bulatovič 2012 [13]	104	≥1 yrs	9.8 mg/m^2^ (NA)	38.5/NA	50/NA
Wallace 2012 [43]	43	5.2 mo (mean)	0.5 mg/kg (s.c.)	NA/23.3	NA/16.3
Moncrieffe2013 [51]	87	1.3 yrs (med)	10–15 mg/m^2^ (70 % p.o.)	56.3/NA	
Pastore 2015 [53]	69	1.0 yrs (med)	15 mg/m^2^ (62 % p.o.)	52.2/NA	
Fraňova 2016	55	3.5 mo (med)	14.2 mg/m^2^ (18 % p.o.)	50.9/30.9	70.9/56.4

*MTX*, Methotrexate

*CID*, Clinical Inactive Disease [40]

*NA*, Not available

^a^Patients were non-responders to the dose of 10 ± 2.3 mg/m2 after the first 6 months

^b^Criteria for inactive disease: Absence of active arthritis and ESR < 20 mm/h

^c^Dose 15 mg/m^2^ orally, non-responders at 3 months switched to 30 mg/m2 s.c

Multiple studies have dealt with potential biomarkers of response to MTX that could inform our therapeutic decision-making and lead to early introduction of other treatments in suspected non-responders, reviewed by Dijkhuizen [5]. Among these high serum concentration of MRP8/14 protein at baseline [51] and long-chain MTX polyglutamates at 3 months of the treatment [52] predicted a favourable response to MTX. From genetic factors single-nucleotide polymorphisms of genes involved in MTX metabolic pathways and some novel candidate genes have been suggested, though their relevance is yet to be confirmed [53–55].

The spectrum of adverse events of MTX therapy has expanded by the recognition and systematic evaluation of so called conditioned response in the form of anticipatory and associative intolerance [34, 35, 56–58]. In this study we have attempted to distinguish toxic adverse events limited to laboratory changes and post-dose GI symptoms from the intolerance featuring GI symptoms around MTX administration (anticipatory) and when thinking about MTX (associative) using a previously validated scoring system MISS [34] (Table 5). In addition, behavioral symptoms at MTX administration were also systematically recorded.

Proportion of patients who featured measurable toxic adverse events of transaminase elevation and/or cytopenia was similar to published series [32, 58–60] and was mild and easy to manage in most cases. Presence of patient-reported GI toxicity as well as intolerance and behavioral symptoms were frequent (Table 4). MTX intolerance (MISS ≥ 6) in at least one visit during the 12 months of treatment was recorded in 45.5 % of our patients. This figure is similar to that reported by Bulatovič and van Dijkhuizen (50.5 % and 41.5 %, respectively) despite the fact that our patients received higher MTX doses (14.2 mg/m^2^ versus 10.2 and 9.9 mg/m^2^, respectively) administered parenterally in most cases [5, 34]. This finding does not support the presence of direct relationship between MTX dose and route of administration and its intolerance. This is in agreement with our previous observation where higher doses of MTX associated with increased intracellular MTX concentrations did not lead to an increased rate of MTX toxicity [15].

Severity of MTX intolerance is difficult to assess due to the subjective nature of patient-reported symptoms. Therefore we believe that an impact of reported adverse events on the treatment itself should serve as the main measure of their clinical significance. Despite the high frequency of complaints in our study they eventually led to MTX withdrawal in 2 patients only. It is our impression that this favourable outcome was associated with the systematic approach to MTX-related complaints in our clinics. MISS form was administered along other patient-reported questionnaires to every patient at routine follow-up visits by the nurse specialist who checked its completion and then discussed with the treating physician who reviewed the ticked items with the patient and/or the accompanying parent and suggested potential solutions tailored to the individual patient characteristics and needs. These discussions covered also the analysis of the risk/benefit ratio of MTX therapy and re-assessment of the future treatment plan. Such a systematic parent/patient education and re-assurance served as an additional component of intolerance management. In the light of the massive evidence on the efficacy and tolerance of biologics, MTX adverse effects are becoming more visible than 20 years ago when therapeutic options for JIA were limited. Moreover, incorporation of patient-reported outcomes into the routine disease assessment has led to better appreciation of an impact of subjective complaints including drug intolerance on the patient and family health-related quality of life.

The relatively short duration of follow-up reported here did not allow for assessment of the sustainability of treatment response as well as the long-term evolution of adverse events. Moreover, direct comparison of treatment efficacy and adverse events between patients treated with oral versus parenteral MTX was not possible due to relatively small numbers of patients, mainly those treated orally. Small patient number also contributed to the inability to detect any significant variables associated with either treatment response or presence of adverse events (Table 6). Although JIA treatment strategy has been established at our Unit the study retained an observational character where therapy was based on the treating physician's decision. Therefore systematic stratification of patients into more homogeneous treatment groups that would improve our ability to analyze treatment variables was not possible.

## Conclusions

As long as the reliable and widely accessible system of prediction of MTX efficacy and intolerance is not available, optimal clinical approach to MTX therapy remains of upmost importance. This study brings additional evidence for the high efficacy of MTX especially in early disease and underlines importance of using the dose around 15 mg/m^2^ administered parenterally in order to evaluate treatment response early in the disease course. Such a treatment strategy does not appear to increase the rate and severity of MTX adverse events when compared to oral treatment using lower doses. Limited by short duration of follow-up and relatively small patient number true significance of our findings has yet to be confirmed through the ongoing extension of the study.

## Abbreviations

ACR Pedi: American College of Rheumatology Pediatric
CID: Clinically Inactive Disease
CHAQ: Childhood Health Assessment Questionnaire
CRP: C-reactive protein
ESR: Erythrocyte Sedimentation Rate
IATH: Intraarticular triamcinolone hexacetonide
JADAS: Juvenile Arthritis Disease Activity Score
JIA: Juvenile Idiopathic Arthritis
MISS: Methotrexate Intolerance Severity Score
MTX: Methotrexate
NSAID: Non-steroidal Anti-Inflammatory Drugs
TNF: Tumour-Necrosis Factor
